# Predictive value of ovarian stroma measurement for cardiovascular risk in polycyctic ovary syndrome: a case control study

**DOI:** 10.1186/1757-2215-3-25

**Published:** 2010-11-09

**Authors:** Giuseppe Loverro, Giovanni De Pergola, Edoardo Di Naro, Massimo Tartagni, Cristina Lavopa, Anna Maria Caringella

**Affiliations:** 1 Clinic of Obstetrics and Gynecology III, University of Bari, School of Medicine, Policlinico, Piazza Giulio Cesare, 70124 Bari, Italy; 2 Section of Internal Medicine, Endocrinology, Andrology and Metabolic Disease. Department of Emergency and Organ Transplantation, University of Bari, School of Medicine, Policlinico, Piazza Giulio Cesare, 70124 Bari, Italy

## Abstract

**Background:**

To verify the feasibility of ovarian stromal evaluation and correlate ovarian parameteres (echogenicity and volume) with hyperandrogenism, and both cardiovascular and metabolic risk factors in PCOS.

**Methods:**

Twenty four young PCOS patients and twelve age-matched control women were enrolled. Diagnosis of PCOS was based on the Rotterdam criteria. Ultrasound ovarian study included ovarian volume, stromal volume, stromal area and stromal area/total ovarian area ratio (S/A). Concerning hormones, insulin, LH, FSH, estradiol, androstenedione, testosterone, DHEAS, 17-hydroxy-progesterone, and SHBG were measured during the early follicular phase (days 2-5). Cardiovascular risk factors were represented by fasting plasma levels of glucose, lipids (total and HDL-cholesterol), plasminogen activator inhibitor 1 (PAI-1), von-Willebrand factor (vWF), and adiponectin. Carotid intima-media thickness (C-IMT) was measured as a parameter of cardiovascular risk.

**Results:**

A positive correlation between the S/A ratio and plasma levels of testosterone (p < 0.05) and androstenedione (p < 0.05) was found. The stromal volume, stromal area and S/A ratio were also significantly and positively correlated with PAI-1, and vWF levels, and with IMT in PCOS women (P < 0.05).

**Conclusions:**

This study shows that the ultrasound measurement of ovarian stroma is a predicting factor of hyperandrogenism degree, prothrombotic factors and cardiovascular risk in patients with PCOS.

## Background

Polycystic ovary syndrome (PCOS) is an endocrine disorder that affects 7-8% of women during reproductive age and is currently considered the most common cause of female infertility [1,2].

Oligo-anovulation, clinical hyperandrogenism, obesity, elevated levels of circulating androgens and LH [2-4], insulin resistance and/or compensatory hyperinsulinemia are features not invariably associated with the syndrome [5].

The strict criteria for diagnosis of PCOS have been long debated, but a recent joint ASRM/ESHRE consensus has proposed a new definition of the syndrome, that includes and emphasizes the morphology of polycystic ovaries [6].

In this definition, at least two of the following three criteria are necessary for diagnosis: 1) oligo- and/or anovulation, 2) hyperandrogenism (clinical and/or biochemical), and 3) the ultrasonic appearance of polycystic ovaries.

Concerning ultrasounds diagnostic criteria of PCOS, these have progressively changed accordingly to technological improvement, evolving from a simple evaluation of the ovarian volume to the identification of a typical follicular pattern and, lastly, to modifications of the ovarian stroma [7].

The presence of 12 or more follicles measuring 2-9 mm in diameter and increased total ovarian volume (>10 cm^3^) are considered satisfying criteria for ultrasonic identification of PCOS, since they have enough specificity and sensitivity [8].

Although various authors have emphasized the diagnostic value of ovarian stroma measurement, this parameters has been confined to the research field. Even though ovarian stromal hypertrophy is largely involved in the pathophysiology of PCOS [6] and it could be properly identified by vaginal endosonography [9], the acceptance of ultrasound ovarian stromal hypertrophy as a additional criterion for diagnosis of PCOS is still controversial. This is due to the fact that results are strictly dependent on both the experience of the operator and the quality of ultrasound machine equipment, with a risk of low reproducibility of ultrasound measurements. By contrast, in our opinion, ultrasound ovarian stromal evaluation may be useful in increasing the predictivity of diagnosis. The sensitivity and specificity of ultrasounds ovarian stromal measurement have been shown to be 94% and 90% respectively, in the diagnosis of polycystic ovaries [10]. Moreover, Venturoli et al have suggested that higher stroma volume or higher stroma area are the most sensitive and specific ultrasonic parameters of PCO [11]. Interestingly, higher stromal volume and echodensity, observed by ultrasounds, have been shown to correspond to prominent theca lutheal cells described by Stein and Leventhal [12], and doubling of cross-sectional ovarian area, observed by ultrasound, has been shown to be expression of a marked increase (33%) of cortical portion, and of a five-fold increase of medullar stroma [13] in PCOS. Lastly, Kyei-Mensah et al have shown that a higher ovarian size in patients with PCO is mainly accounted for by the differences in stromal volume, with no significant changes in the follicular/cortical volume [14]. Many parameters to judge the ovarian morphological alterations has been adopted: doubling of the cross-sectional area, doubling of the number of ripening and atresic follicles, a 50% increase in tunica thickness, a 33% increase of cortical and a five-fold increase of medullar stroma [13].

The present study, performed in young and never treated nulliparous PCOS women, has been addressed to evaluate the possible routinary clinical feasibility of ultrasounds ovarian stromal evaluation and its correlation with androgens, insulin resistance (HOMA test), and instrumental and metabolic makers of early atherosclerosis.

## Methods

### Subjects

Women were enrolled according to specific inclusion criteria in order to provide a homogeneous group of patients. Inclusion criteria were the diagnosis of PCOS, the absence of previous pregnancy or previous hormone or any other medical treatment and age ≤ 27 years. Diagnosis of PCOS was based on the presence of two out of three criteria second revised Rotterdam consensus [6]: unilateral or bilateral polycystic ovaries (PCO) on transvaginal scan, clinical or biochemical features of hyperandrogenism and menstrual irregularity (chronic anovulation with amenorrhea or chronic oligoamenorrhea).

Hyperandrogenaemia was defined on the basis of high serum androgen levels, mainly testosterone (≥0.6 ng/ml) and androstenedione (≥3.0 ng/ml). Clinical hyperandrogenism was defined by the presence of hirsutism. This was assessed by the Ferriman-Gallwey-Lorenzo scores, with patients having scores ≥8 being considered as hirsute.

Exclusion criteria were smoking habit, no ovarian causes of hyperandrogenism (adrenal enzymatic deficiencies, Cushing's syndrome and tumors), manifest diabetes mellitus, hyperprolactinemia, any respiratory or cardiovascular disease, hypertension, and any hormonal or drug treatment (including oral contraceptives) over the 6 months before the study.

On this basis, twenty four women with PCOS were enrolled consecutively at the Outpatient Clinic of Obstetrics and Gynecology Department, University of Bari, School of Medicine.

Twelve age-matched healthy volunteer women were examined as control group. They had normal ovaries, no evidence of hyperandrogenemia (hirsutism), and regular menstrual cycles, as well, they had never been treated for menstrual disturbances, infertility or hirsutism at any time.

The study was approved by the Institutional Review Board of the Bari University Hospital, and informed consent was obtained from all women before entry into the study. The investigation was performed according to the principles expressed in the "Declaration of Helsinki".

#### Anthropometric measurements and general data

Weight was measured to the nearest kg. Height was determined to the nearest meter (m). BMI was calculated as the weight (kg) divided by the square of height (m). The minimum waist measurements between the pelvic brim and the costal margin, and the maximum hip measurement at 5° the level of the greater trocanters, were used to calculate the waist/hip ratio (WHR). Systolic and diastolic blood pressure was evaluated in three different occasions in the right arm with the subjects in a relaxed sitting position in all patients and controls. All women under study were normotensive.

#### Hormonal and metabolic parameters

After an overnight fast, blood samples were drawn at 8 a.m. during the early follicular phase (cycle day 2, 3, 4 or 5) to measure the plasma levels of hormones, glucose, lipids and other metabolic and cardiovascular risk factors. Hormonal study included insulin, LH, FSH, estradiol, androstenedione (A), testosterone (T), dehydroepiandrosterone sulphate (DHEAS), 17-hydroxy-progesterone (17-OHP), sex hormone-binding globulin (SHBG) and prolactin (PRL). Plasma LH, FSH, estradiol, T, A, DHEAS, 17-OHP and PRL concentrations were measured by using a recombinant immunoassay, whereas SHBG was measured using a RIA. For all measurements, commercial kits were used (Diagnostic System Laboratories, Inc., Webster. TX), with intra- and interassay coefficients of variation <10%.

Plasma insulin concentrations were measured by radioimmunoassay (Behring, Scoppitto, Italy) and intra- and inter-assay coefficients of variation were 3.7% and 7.5% respectively. Plasma glucose levels were determined by the glucose-oxidase method (Sclavo, Siena, Italy). Plasma adiponectin was measured by a specific RIA obtained from Linco Research, Inc. (St. Charles, MO), with minor modifications. Recombinant human adiponectin was used as standard, and a multispecies adiponectin rabbit antiserum was used. The assay buffer contained 10.0 mmol phosphate buffer, pH 7.6, sodium azide (0.09%) and BSA (0.15%). The intra- and interassay coefficients of variation were 3.3% and 8.4%, respectively. Insulin resistance was assessed by using the homeostasis model assessment (HOMA_IR_), based on a mathematical correlation between fasting plasma glucose and insulin levels [15].

#### Prothrombotic parameters

Subjects lay down in the supine position for 20 min before blood collection. Blood samples for PAI-1 antigen and vonWillebrand factor antigen (vWF) were kept on crushed ice until centrifugation. Samples were centrifuged at 2500 × g for 15 min at 4°C within 15 min after blood collection. The resulting plasma was stored in small aliquots in a -70°C freezer until assay. PAI-1 antigen and vWF concentrations were determined as previously described [16]. Both intra-assay and inter-assay coefficients of variation in all the above methods were less than 7.5%

#### Ultrasound ovarian parameters

US examinations were performed in the early follicular phase (days 1 ± 3) of the menstrual cycle, when the ovaries are relatively quiescent by a single operator following the criteria of the Rotterdam ESHRE/ASRM Consensus, 2004 [6]. Transvaginal US (TVUS) was performed on each patient using a 6.5 MHz transvaginal transducer (Aloka ALPHA 10 PROSOUND (Aloka, Tokyo, Japan): ovarian volume, ovarian area, stromal area, S/A ratio and the number, diameters and distribution of follicles were recorded.

The transabdominal route was not used. In fact, it has been argued that transvaginal ultrasound is a more sensitive method for the detection of polycystic ovaries.

In the TVUS data used for statistical analysis were the mean of observed values for the left and right ovaries.

It was identified each ovary and measured the maximum diameter in each of three planes (longitudinal, antero-posterior and transverse).

Ovarian volume (OV) was calculated for each ovary using the formula for a prolate ellipsoid: π/6 × (D1 × D2 × D3), where D represented the maximum diameter in transverse, antero-posterior and long section. The ovarian area (OA) and stromal area (SA) were performed using the formula for an ellipse (length × width × π/4) and following the mean stroma/total area (S/A) ratio was obtained.

Noteworthy, the reproducibility of the stromal volume measurement was very similar to that of the ovarian volume.

The mean of the stroma of the two ovaries was used for each patient in statistical analyses

#### Evaluation of common carotid artery IMT

Ultrasonographic studies of common carotid arteries were performed bilaterally by a single observer. The value of common carotid arteries that was considered for statistical analyses was the mean of right and left measurements. All studies were performed with a Hewlett Packard Sonos 1500 (Hewlett Packard, Avondale, PA, USA) using a 7.5 MHz high-resolution probe. IMT was defined as a low-level echo grey band that does not project into the arterial lumen. It was measured during end-diastole as the distance from the leading edge of the second echogenic line of the far walls of the distal segment of the common carotid artery, the carotid bifurcation, and the initial tract of internal carotid artery on both sides. Measurements were performed 0.5, 1, and 2 cm below the bifurcation (three measurements on each side), and the average measurement was taken as the IMT. IMT measurements were always performed in plaque-free arterial segments.

### Statistics

Results are presented as mean ± standard deviation (SD) for all parameters. Variables with a skewed distribution (waist circumference, insulin, HOMA_IR_, PAI-1) were logarithmically transformed before analyses, to improve the approximation to a Gaussian distribution. Student's t-test for independent samples was used to evaluate the differences between groups.

*P *values < 0.05 were considered statistically significant. All statistical analyses were performed using the STATISTICA 6.0 for Windows, StatSoft Inc. (1995) software (Tulsa, OK, USA).

## Results

Table 1 shows general, anthropometric (age, BMI WHR) data of PCOS and control women. PCOS patients presented a significantly higher BMI, WHR (P < 0.05).

**Table 1:** General anthropometric in PCOS and control women

	PCOS	Control	
	N = 24	N = 12	*P-value*
**Age (years)**	22.87 ± 4.28	21.66 ± 5.15	**P = 0.46**
**BMI (weight/m2)**	31 ± 7.0	25.16 ± 6.97	P = 0.02^a^
**Waist/Hip ratio (WHR)**	0.87 ± 0.15	0.84 ± 0.12	P = 0.00^a^

Values are mean ± SD;

^a ^P < 0.001 (Student's t test) PCOS vs control group.

Plasma concentrations of vWF, fasting blood glucose, total cholesterol were not significantly different between the two groups (P = 0.15; P = 0.17; P = 0.17), whereas the levels of fasting insulin, HOMA_IR_, colesterol HDL and PAI-were higher in PCOS patients (P < 0.05). Noteworthy, the level of serum adiponectin were lower in PCOS women in respect to the control (P = 0.001) (Table 2).

**Table 2:** Metabolic and cardiovascular risk parameters in control women and in women with PCOS

	PCOS	Control	
	N = 24	N = 12	*P-value*
**Fasting blood glucose (mg/dl)**	86.29 ± 9.90	82.08 ± 5,12	**P = 0.17**
**Fasting insulin (mUI/ml)**	22.0 ± 12.0	7.65 ± 5.48	P = <0.001^a^
**HOMA_IR_**	4.54 ± 2.77	1.82 ± 1.17	P = <0.05^a^
**Total cholesterol (mg/ml)**	183.4 ± 30.35	168.6 ± 29.30	**P **= **0.17**
**HDL cholesterol (mg/ml)**	51 ± 8,67	59,16 ± 9,19	P = <0.05 ^a^
**PAI-1 (ng/ml)**	37,80 ± 20,98	17.2 ± 18.2	P = 0.007^a^
**F von Willebrand (%)**	86,86 ± 34,25	103,65 ± 27,75	**P = 0.15**
**Adiponectin**	6,05 ± 3,84	24 ± 4,26	P = 0.000^a^

Values are mean ± SD;

^a ^P < 0.001 (Student's t test) PCOS vs control group.

The levels of plasma FSH, estradiol, and 17 OHP did not differ (P = 0.52; 0.86; 0.15) between the groups. The LH, Androstenedione, Testosterone, DHEA-S and SHBG concentration was significantly higher in PCOS (P < 0.05) (Table 3).

**Table 3:** Sex hormone levels in control women and in patients with PCOS

	PCOS	Control	
	N = 24	N = 12	*P-value*
**FSH (U/l)**	4,77 ± 1,24	4,47 ± 1,48	**P = 0.52**
**LH (U/l)**	5,61 ± 3,44	3,17 ± 1,26	P = 0.02^a^
**E2 (pg/ml)**	41 ± 35,56	39,05 ± 22,38	**P =0.86**
**A (ng/ml)**	3,09 ± 1,33	1.67 ± 0.83	P = 0.002 ^a^
**T (ng/ml)**	0,65 ± 0,50	0.22 ± 0.08	P = 0.006 ^a^
**SHBG (nmol/l)**	44,05 ± 2,27	54,55 ± 19,40	P = 0.01 ^a^
**DHEA-S (mg/m)**	2,19 ± 0,69	1.61 ± 0.96	P = 0.04 ^a^
**17 0H P (ng/ml)**	1,54 ± 1,87	0.74 ± 0.53	**P = 0.15**

Values are mean ± SD;

^a ^P < 0.001 (Student's t test) PCOS vs control group.

Analysis of the ultrasound appearance of the ovaries showed that PCOS patients showed had a higher ovarian and stromal volume, stromal area and a higher area stromal area/area ovary ratio (S/A ratio) compared with the control women (P < 0.05)(Table 4).

**Table 4:** Ovarian ultrasound measurements in PCOS and control women

	Control	PCOS	
	N = 12	N = 24	*P-value*
**Ovarian Volume (cm^3^)**	4.98 ± 2.91	9.13 ± 3.98	P = <0.01^a^
**Ovarian Area (cm^2^)**	41.3 ± 16.3	47.4 ± 17.4	**P = 0.31**
**Stromal Volume (cm^3^)**	0.59 ± 0.66	11.4 ± 4.57	P = <0.001 ^a^
**Stromal Area (cm^2^)**	8.12 ± 5.46	13.9 ± 6.10	P = <0.01 ^a^
**S/A ratio**	4.37 ± 1.58	7.49 ± 4.45	P = <0.05 ^a^

Values are mean ± SD;

S/A ratio = ratio between the stromal and the total ovarian area

^a ^P < 0.001 (Student's t test) PCOS vs control group

The total ovarian area was not significantly different between the two groups.

The correlation between ovary ultrasound measurement and anthropometric/metabolic parameters has been performed in PCOS subjects and control women in order to clarify the clinical significance of the above specified ultrasound measurement (Table 5).

**Table 5:** Linear correlation (Rho-Spearman) between TVUS findings and hormonal and metabolic plasma levels.

	OV (cm3)	OA (cm2)	SV (cm3)	SA (cm2)	S/A ratio
**BMI (weight/m2)**	P = 0.02^a^	P = 0.45	P = 0.31	P = 0.37	P = 0.84
**Waist/Hip ratio (WHR)**	P = 0.01 ^a^	P < 0.01 ^a^	P = 0.77	P = 0.07	P = 0.77
**HOMA test**	P = 0.14	P = 0.14	P = 0.17	P = 0.53	P = 0.43
**FSH (U/l)**	P = 0.73	P = 0.87	P = 0.72	P = 0.58	P = 0.72
**LH (U/l)**	P = 0.24	P = 0.51	P = 0.62	P = 0.31	P = 0.13
**A (ng/ml)**	P = 0.66	P = 0.15	P = 0.55	P = 0.81	P < 0.05 ^a^
**T (ng/ml)**	P = 0.76	P = 0.14	P = 0.97	P = 0.74	P = 0.03 ^a^
**SHBG (nmol/l)**	P = 0.94	P = 0.43	P = 0.38	P = 0.83	P = 0.77
**DHEA-S (mg/m)**	P = 0.44	P = 0.35	P = 0.82	P = 0.89	P = 0.64
**17 0H P (ng/ml)**	P = 0	P = 0.56	P = 0.78	P = 0.31	P = 0.19
**Cholesterol (mg/dl)**	P < 0.05 ^a^	P = 0.17	P = 0.92	P = 0.77	P = 0.56

^a ^P < 0.001 (Correlation and regression linear) PCOS vs control group

A significant correlation has been found between the ultrasound measurements and some biochemical and anthropometric characteristics. In fact, we observed the direct association between ovarian volume and BMI (p < 0.05), WHR (p < 0.01), and total cholesterol (p < 0.05), as well between the ovarian area and WHR (p < 0.01).

As endocrine point of view, a positive correlation between the S/A ratio and the plasma levels of testosterone (p < 0.05) and androstenedione (p < 0.05) has been found.

An important finding was a linear relationship between the ultrasound parameters of the stroma and some cardiovascular risk factors (Table 6)

**Table 6:** Linear correlation (Rho-Spearman) between TVUS findings and cardiovascular risck factors in PCOS group.

	OV (cm3)	OA (cm2)	SV (cm3)	SA (cm2)	S/A ratio
**PAI-1 (ng/ml)**	P = 0.29	P = 0.21	P = 0.000 ^a^	P = 0.000 ^a^	P = 0.000 ^a^
**F von Willebrand (%)**	P = 0.15	P = 0.31	P = 0.000 ^a^	P = 0.000 ^a^	P = 0.000 ^a^
**Adiponectine**	P = 0.45	P = 0.21	P = 0.001 ^a^	P = 0.000 ^a^	P = 0.08
**IMT**	P = 0.000 ^a^	P = 0.000 ^a^	P = 0.007	P = 0.000 ^a^	P = 0.000 ^a^

^a ^P < 0.001 (Correaltion and regression linear) PCOS vs control group

In fact, stromal volume, stromal area and S/A ratio are significantly and positively correlated, in PCOS women, with the level of PAI-1, von Willebrand factor and with intima media thickness (IMT).

The IMT of carotid artery demonstrated a significant linear correlation with not only stromal US parameters but also with ovarian volume and area evaluation.

The levels of adiponectin were significantly correlate with stromal measurement, mainly with S/A ratio.

None of total ovarian ultrasound measurements (ovarian volume, ovarian area) demonstrated, in PCOS patient, any positively correlation with this cardio metabolic risk factor.

## Discussion

The present study, performed in untreated young nulliparas women affected by PCOS, was addressed to evaluate the possible associations between ovarian stroma, measured by ultrasounds, and hormones, and metabolic and cardiovascular risk parameters.

PCO patients showed significantly higher ovarian volume, stroma volume, stroma area and S/A ratio as compared to the control group, confirming the diagnostic role of ultrasounds stroma measurement in the evaluation of PCOS patients [17]. This is an important result, since Joint ASRM/ESHRE consensus meeting on PCOS established to take into account only the presence of 12 or more follicles measuring 2 ± 9 mm in diameter, and/or the increased ovarian volume (>10 cm3), for the diagnosis of PCOS. Noteworthy, although ovarian volume is more reliable in routinary clinical practice, only ovarian stroma measurement may correspond to histological findings of prominent theca and fibrotic thickening of prominent lutheal cell albuginea, alterations that explain many of clinical features of the syndrome [18].

We found a significant correlation between ultrasonographic S/A ratio and both testosterone and androstenedione serum levels. These results are in line with previous studies showing that only ultrasound ovarian stroma has a positive correlation with hyperadrogenic status and androgens levels [7-18]. Therefore, evaluation of the S/A ratio may enhance the predictive ability of ultrasounds to identify women with PCOS, thus differentiating polycystic and multifollicular ovaries, and reducing the risk of false-positive or negative cases. For istance, we could not find a significant correlation between androgen levels and total ovarian area or ovarian volume, suggesting that ovarian volume is mainly influenced by the number of follicles, whereas it is not expression of PCOS endocrine impairment.

In this study, reproducibility of the measurements of stromal volume was very close to that of ovarian volume and of cortical follicular count; therefore, we strongly believe that ultrasound determination of ovarian stroma volume might be routinely used in clinical practice, at least whether a modern ultrasound machine is used, togheter to the determination of serum androgens or the evaluation of Ferriman-Gallwey-Lorenzo scores.

Concerning the association of ultrasounds ovary parameters and cardiovascular risk variables, stroma volume, stroma area, and S/A ratio showed a significant association with well known cardiovascular risk factors such as plasma levels of adiponectin, PAI-1 and vWF. Moreover, stroma parameters showed a significant correlation with CCA-IMT, that is a well known early sign of atherosclerosis. All these results confirm several studies showing that young PCOS women have an adverse cardiovascular risk profile, and a higher cardiovascular risk [19,20]. Previous studies had clearly shown an association between PCOS and carotid intima-media thickness [21] or anteroposterior diameter of infrarenal abdominal aorta [22]. Moreover, other studies had shown a correlation between PCOS and serum markers of atherosclerosis such as CRP [23], interleukin-18 [24], homocysteine [25], and endothelial dysfunction [26,27]. These data suggest a new role of ultrasound stroma measurement in the evaluation of the cardiovascular risk in young patients affected by PCOS, who do not show clinical signs of cardiovascular disease.

## Conclusions

In conclusion, this study shows that ultrasound measurement of ovarian stroma is useful in predicting hyperandrogenism severity and cardiovascular risk in women affected by PCOS. In particular, the ratio between stroma and total ovarian area is associated with higher androgen serum levels, thus improving the diagnostic accuracy of PCOS, whereas the stroma itself is related to the intima-media thickness of common carotid artery and the plasma levels of important prothrombotic factors such as PAI-1 and vWF. All these data suggest a possible role of ultrasounds stroma measurement in the diagnosis of PCOS and in the quantification of cardiovascular risk in young patients affected by PCOS.

## Competing interests

The authors declare that they have no competing interests.

## Authors' contributions

GL conceived of the study, and participated in its design and coordination and helped to draft the manuscript. He have also given final approval of the version to be published.

GP have made substantial contributions to acquisition of data.

EDN have made substantial contributions to analysis and interpretation of data;

MT participated in the design of the study and performed the statistical analysis.

CL have made substantial contributions to acquisition of data,

AMC have made substantial contributions to interpretation of data

All authors read and approved the final manuscript.
